# Correction: Single-item versus scale: Comparing respondent demographic, social, and health characteristics by measure of loneliness using the Canadian Longitudinal Study on Aging (CLSA) data

**DOI:** 10.1371/journal.pone.0346161

**Published:** 2026-03-27

**Authors:** Stephanie A. Chamberlain, Lauren E. Griffith, Rachel D. Savage, Jesse Batara, Wenting Yan, Andrea Gruneir

The Acknowledgement section for this article is incorrect. The correct Acknowledgement section is as follows: This research was made possible using the data/biospecimens collected by the Canadian Longitudinal Study on Aging (CLSA). Funding for the Canadian Longitudinal Study on Aging (CLSA) is provided by the Government of Canada through the Canadian Institutes of Health Research (CIHR) under grant reference: LSA 94473 and the Canada Foundation for Innovation, as well as the following provinces, Newfoundland, Nova Scotia, Quebec, Ontario, Manitoba, Alberta, and British Columbia. This research has been conducted using the CLSA dataset Baseline Tracking Dataset version 3.6, Baseline Comprehensive Dataset version 4.2, Follow-up 1 Tracking version 2.1, and Follow-up 1 Comprehensive version 3.0 under Application Number 20CA004. The CLSA is led by Drs. Parminder Raina, Christina Wolfson and Susan Kirkland. The time and commitment of the participants to the CLSA study platform is gratefully acknowledged, without whom this research would not be possible.
