# Supplementary material for: Single-item versus scale: Comparing respondent demographic, social, and health characteristics by measure of loneliness using the Canadian Longitudinal Study on Aging (CLSA) data
Source: PLoS One. 2026 Feb 4;21(2):e0341572. doi: 10.1371/journal.pone.0341572 (PMC12871960; doi:10.1371/journal.pone.0341572)
Supplement: S1 Table — (DOCX) [file pone.0341572.s001.docx]

SI Table. Description of the measures of loneliness in the Canadian Longitudinal Study on Aging (CLSA) follow-up 1 survey

| **Construct/Scale** | **Items** | **Response categories** | **Min-Max score** | **Coefficient (alpha)** | **Mean** | **SD*** | **Median** |
| --- | --- | --- | --- | --- | --- | --- | --- |
| 3-item Loneliness Scale | “How often do you feel left out?”  “How often do you feel isolated from others?”  “How often do you feel that you lack companionship?” | 1: Hardly ever  2: Some of the time  3: Often | 3-9 | 0.763 | 3.861 | 1.331 | 4.0 |
| Single item | “How often did you feel lonely?” | 1: Rarely or never (less than one day)  2: Some of the time (1-2 days)  3: Occasionally (3-4 days)  4: All the time (5-7 days) | 1-4 | N/A | 1.365 | 0.734 | 1.0 |

^*SD=Standard Deviation^
