# Supplementary material for: Single-item versus scale: Comparing respondent demographic, social, and health characteristics by measure of loneliness using the Canadian Longitudinal Study on Aging (CLSA) data
Source: PLoS One. 2026 Feb 4;21(2):e0341572. doi: 10.1371/journal.pone.0341572 (PMC12871960; doi:10.1371/journal.pone.0341572)
Supplement: S2 Table — (DOCX) [file pone.0341572.s002.docx]

S2 Table. Descriptive characteristics of CLSA Follow-up 1 survey respondents by dichotomized loneliness measure for 3-item scale and single-item

| **Variables** | **Single Item** | | | **3-item Scale** | | |
| --- | --- | --- | --- | --- | --- | --- |
|  | **Lonely** | **Not Lonely** | **Total** | **Lonely** | **Not Lonely** | **Total** |
|  | **N (%)** | **N (%)** | **N (%)** | **N (%)** | **N (%)** | **N (%)** |
| **Total** | 10205(24) | 33179(76) | 43384(100) | 10246(23) | 34127(77) | 44373(100) |
| **Age** |  |  |  |  |  |  |
| 46-55 | 1659(16) | 6076(18) | 7735(18) | 1828(18) | 6099(18) | 7927(18) |
| 56-65 | 3303(32) | 11163(34) | 14466(33) | 3454(34) | 11287(33) | 14741(33) |
| 66-75 | 2715(27) | 9606(29) | 12321(28) | 2688(26) | 9859(29) | 12547(28) |
| 76-85 | 2124(21) | 5555(17) | 7679(18) | 1922(19) | 6009(18) | 7931(18) |
| 86+ | 404(4) | 779(2) | 1183(3) | 354(3) | 873(3) | 1227(3) |
| **Age (Mean, SD)** | 66.9(10.0) | 65.7(10.0) | 65.9(10.2) | 66.2(10.5) | 65.9(10.1) | 66.0(10.2) |
| **Age median (Q1-Q3)** | 58-75 | 57-73 | 58-73 | 58-74 | 58-73 | 58-73 |
| **Gender** |  |  |  |  |  |  |
| Gender diverse | 14(0.14) | 24(0.07) | 38(0.09) | 11(0.11) | 26(0.08) | 37(0.08) |
| Women | 5795(57) | 16365(49) | 22160(51) | 5756(56) | 16921(50) | 22677(51) |
| Men | 4392(43) | 16779(51) | 21171(49) | 4475(44) | 17170(50) | 21645(49) |
| **Education** |  |  |  |  |  |  |
| Less than university | 5560(54) | 16705(50) | 22265(51) | 5551(54) | 17252(51) | 22803(51) |
| University or higher | 3801(37) | 14743(44) | 18544(43) | 3853(38) | 15060(44) | 18913(43) |
| **Ethnicity** |  |  |  |  |  |  |
| All else | 474(5) | 1106(3) | 1580(4) | 483(5) | 1140(3) | 1623(4) |
| White | 9731(95) | 32073(97) | 41804(96) | 9763(95) | 32987(97) | 42750(96) |
| **Geographic region** |  |  |  |  |  |  |
| Rural | 954(9) | 3609(11) | 4563(11) | 1012(10) | 3618(11) | 4630(10) |
| Urban | 9251(91) | 29570(89) | 38821(89) | 9234(90) | 30509(89) | 39743(90) |
| **Income** |  |  |  |  |  |  |
| <$20,000 | 930(9) | 1077(3) | 2007(5) | 976(10) | 1085(3) | 2061(5) |
| $20,000-<$50,000 | 3118(31) | 6523(20) | 9641(22) | 3096(30) | 6790(20) | 9886(22) |
| $50,000+ | 5282(52) | 23612(71) | 28894(67) | 5310(52) | 24181(71) | 29491(66) |
| **Marital status** |  |  |  |  |  |  |
| Single, never married | 1400(14) | 2363(7) | 3763(9) | 1461(14) | 2375(7) | 3836(9) |
| Divorced/separated | 2037(20) | 3223(10) | 5260(12) | 2074(20) | 3310(10) | 5384(12) |
| Married/common law | 4637(45) | 25021(75) | 29658(68) | 4912(48) | 25365(74) | 30277(68) |
| Widowed | 2127(21) | 2555(8) | 4682(11) | 1795(18) | 3059(9) | 4854(11) |
| **Living alone** |  |  |  |  |  |  |
| No | 5659(55) | 27171(82) | 32830(76) | 5950(58) | 27214(80) | 33164(75) |
| Yes | 4546(45) | 6008(18) | 10554(24) | 4296(42) | 6913(20) | 11209(25) |
| **Number of chronic conditions** |  |  |  |  |  |  |
| <4 | 7244(71) | 27361(82) | 34605(80) | 7244(71) | 28348(83) | 35592(80) |
| 4+ | 2961(29) | 5818(18) | 8779(20) | 3002(29) | 5779(17) | 8781(20) |
| **Functional impairment** |  |  |  |  |  |  |
| None | 7718(76) | 28501(86) | 36219(83) | 7669(75) | 29317(86) | 36986(83) |
| Mild/moderate/severe/total | 2143(21) | 3696(11) | 5839(13) | 2258(22) | 3800(11) | 6058(14) |
| **Self-rated mental health** |  |  |  |  |  |  |
| Poor | 250(2) | 124(0.37) | 374(1) | 271(3) | 117(0.34) | 388(1) |
| Fair/Good/Very Good/Excellent | 9935(97) | 33019(100) | 42954(99) | 9948(97) | 33978(100) | 43926(99) |
| **Number of depressive symptoms** |  |  |  |  |  |  |
| <10 | 525(5) | 238(1) | 763(2) | 457(4) | 324(1) | 781(2) |
| 10+ | 9680(95) | 32940(99) | 42620(98) | 9512(93) | 33020(97) | 42532(96) |
| **Number of social contacts** |  |  |  |  |  |  |
| High contact (4-5) | 4561(45) | 17304(52) | 21865(50) | 4224(41) | 17857(52) | 22081(50) |
| Moderate contact (2-3) | 4690(46) | 13853(42) | 18543(43) | 4804(47) | 13919(41) | 18723(42) |
| Low contact (0-1) | 954(9) | 2021(6) | 2975(7) | 1218(12) | 2350(7) | 3568(8) |
| **Number of social activities** |  |  |  |  |  |  |
| High participation (4-5) | 1855(18) | 7264(22) | 9119(21) | 1656(16) | 7551(22) | 9207(21) |
| Moderate participation (2-3) | 5904(58) | 20613(62) | 26517(61) | 5761(56) | 21017(62) | 26778(60) |
| Low participation (0-1) | 2429(24) | 5275(16) | 7704(18) | 2646(26) | 5122(15) | 7768(18) |
| **Anxiety** |  |  |  |  |  |  |
| No | 8638(85) | 30708(93) | 39346(91) | 8389(82) | 30954(91) | 39343(89) |
| Yes | 1534(15) | 2434(7) | 3968(9) | 1575(15) | 2387(7) | 3962(9) |
| **Family doctor in last 12 months** |  |  |  |  |  |  |
| No | 789(8) | 3065(9) | 3854(9) | 851(8) | 3081(9) | 3932(9) |
| Yes | 9394(92) | 30042(91) | 39436(91) | 9364(91) | 30977(91) | 40341(91) |
| **Unmet need** |  |  |  |  |  |  |
| Yes | 1288(13) | 2353(7) | 3641(8) | 1471(14) | 2258(7) | 3729(8) |
| No | 8893(87) | 30770(93) | 39663(91) | 8740(85) | 31818(93) | 40558(91) |
| **Emergency department visit** |  |  |  |  |  |  |
| Yes | 2738(27) | 6936(21) | 9674(22) | 2787(27) | 7177(21) | 9964(22) |
| No | 7436(73) | 26167(79) | 33603(77) | 7420(72) | 26874(79) | 34294(77) |
| **Care received** |  |  |  |  |  |  |
| No care received | 687(7) | 1139(3) | 1826(4) | 737(7) | 1193(3) | 1930(4) |
| Non-professional received | 539(5) | 948(3) | 1487(3) | 555(5) | 982(3) | 1537(3) |
| Professional received | 1311(13) | 3392(10) | 4703(11) | 1390(14) | 3444(10) | 4834(11) |
| Both non-professional and professional received | 7668(75) | 27697(83) | 35365(82) | 7562(74) | 28507(84) | 36069(81) |
