# Supplementary material for: Single-item versus scale: Comparing respondent demographic, social, and health characteristics by measure of loneliness using the Canadian Longitudinal Study on Aging (CLSA) data
Source: PLoS One. 2026 Feb 4;21(2):e0341572. doi: 10.1371/journal.pone.0341572 (PMC12871960; doi:10.1371/journal.pone.0341572)
Supplement: S3 Table — (DOCX) [file pone.0341572.s003.docx]

S3 Table. Descriptive characteristics of CLSA Follow-up 1 survey respondents by loneliness severity (single item and 3-item scale)

| **Variables** | **Single Item** | | | | **3-item Scale** | | | |
| --- | --- | --- | --- | --- | --- | --- | --- | --- |
|  | **Severely Lonely** | **Moderately Lonely** | **Not Lonely** | **Total** | **Severely Lonely** | **Moderately Lonely** | **Not Lonely** | **Total** |
|  | **N (%)** | **N (%)** | **N (%)** | **N (%)** | **N (%)** | **N (%)** | **N (%)** | **N (%)** |
| **Total** | 4618(11) | 5587(13) | 33179(76) | 43384(100) | 5463(12) | 12717(29) | 26193(59) | 44373(100) |
| **Age** |  |  |  |  |  |  |  |  |
| 46-55 | 623(13) | 1036(19) | 6076(18) | 7735(18) | 993(18) | 2212(17) | 4722(18) | 7927(18) |
| 56-65 | 1401(30) | 1902(34) | 11163(34) | 14466(33) | 1909(35) | 4090(32) | 8742(33) | 14741(33) |
| 66-75 | 1274(28) | 1441(26) | 9606(29) | 12321(28) | 1393(25) | 3516(28) | 7638(29) | 12547(28) |
| 76-85 | 1096(24) | 1028(18) | 5555(17) | 7679(18) | 964(18) | 2501(20) | 4466(17) | 7931(18) |
| 86+ | 224(5) | 180(3) | 779(2) | 1183(3) | 204(4) | 398(3) | 625(2) | 1227(3) |
| **Age (Mean, SD)** | 68.2(10.6) | 65.9(10.5) | 65.7(10.0) | 65.9(10.2) | 65.9(10.4) | 66.5(10.4) | 65.8(10.0) | 66.0(10.2) |
| **Age median (Q1-Q3)** | 67(60-70) | 65(57-74) | 65(57-73) | 58-73 | 65.0(57-73) | 66.0(58-74) | 65.0(57-73) | 58-73 |
| **Gender** |  |  |  |  |  |  |  |  |
| Gender diverse | 4(0.1) | 10(0.2) | 24(0.1) | 38(0.1) | 6(0.1) | 13(0.1) | 18(0.1) | 37(0.1) |
| Women | 2668(58) | 3127(56) | 16365(49) | 22160(51) | 3152(58) | 6955(55) | 12570(48) | 22677(51) |
| Men | 1942(42) | 2450(44) | 16779(51) | 21171(49) | 2303(42) | 5745(45) | 13597(52) | 21645(49) |
| **Education** |  |  |  |  |  |  |  |  |
| Less than university | 2604(56) | 2956(53) | 16705(50) | 22265(51) | 3036(56) | 6638(52) | 13129(50) | 22803(51) |
| University or higher | 1555(34) | 2246(40) | 14743(44) | 18544(43) | 1942(36) | 5220(41) | 11751(45) | 18913(43) |
| **Ethnicity** |  |  |  |  |  |  |  |  |
| All else | 188(4) | 286(5) | 1106(3) | 1580(4) | 289(5) | 469(4) | 865(3) | 1623(4) |
| White | 4430(96) | 5301(95) | 32073(97) | 41804(96) | 5174(95) | 12248(96) | 25328(97) | 42750(96) |
| **Geographic region** |  |  |  |  |  |  |  |  |
| Rural | 445(10) | 509(9) | 3609(11) | 4563(11) | 534(10) | 1252(10) | 2844(11) | 4630(10) |
| Urban | 4173(90) | 5078(91) | 29570(89) | 38821(89) | 4929(90) | 11465(90) | 23349(89) | 39743(90) |
| **Income** |  |  |  |  |  |  |  |  |
| <$20,000 | 523(11) | 407(7) | 1077(3) | 2007(5) | 690(13) | 701(6) | 670(3) | 2061(5) |
| $20,000-<$50,000 | 1522(33) | 1596(29) | 6523(20) | 9641(22) | 1733(32) | 3368(26) | 4785(18) | 9886(22) |
| $50,000+ | 2159(47) | 3123(56) | 23612(71) | 28894(67) | 2556(47) | 7744(61) | 19191(73) | 29491(66) |
| **Marital status** |  |  |  |  |  |  |  |  |
| Single, never married | 615(13) | 785(14) | 2363(7) | 3763(9) | 900(16) | 1457(11) | 1479(6) | 3836(9) |
| Divorced/separated | 985(21) | 1052(19) | 3223(10) | 5260(12) | 1266(23) | 2041(16) | 2077(8) | 5384(12) |
| Married/common law | 1858(40) | 2779(50) | 25021(75) | 29658(68) | 2302(42) | 7226(57) | 20749(79) | 30277(68) |
| Widowed | 1159(25) | 968(17) | 2555(8) | 4682(11) | 993(18) | 1985(16) | 1876(7) | 4854(11) |
| **Living alone** |  |  |  |  |  |  |  |  |
| No | 2303(50) | 3356(60) | 27171(82) | 32830(76) | 2907(53) | 8320(65) | 21937(84) | 33164(75) |
| Yes | 2315(50) | 2231(40) | 6008(18) | 10554(24) | 2556(47) | 4397(35) | 4256(16) | 11209(25) |
| **Number of chronic conditions** |  |  |  |  |  |  |  |  |
| <4 | 3083(67) | 4161(74) | 27361(82) | 34605(80) | 3682(67) | 9800(77) | 22110(84) | 35592(80) |
| 4+ | 1535(33) | 1426(26) | 5818(18) | 8779(20) | 1781(33) | 2917(23) | 4083(16) | 8781(20) |
| **Functional impairment** |  |  |  |  |  |  |  |  |
| None | 3357(73) | 4361(78) | 28501(86) | 36219(83) | 3906(71) | 10297(81) | 22783(87) | 36986(83) |
| Mild/moderate/severe/total | 1113(24) | 1030(18) | 3696(11) | 5839(13) | 1375(25) | 2048(16) | 2635(10) | 6058(14) |
| **Self-rated mental health** |  |  |  |  |  |  |  |  |
| Poor | 164(4) | 86(2) | 124(0) | 374(1) | 227(4) | 93(1) | 68(0.3) | 388(1) |
| Fair/Good/Very Good/Excellent | 4440(96) | 5495(98) | 33019(100) | 42954(99) | 5220(96) | 12607(99) | 26099(100) | 43926(99) |
| **Number of depressive symptoms** |  |  |  |  |  |  |  |  |
| <10 | 392(8) | 133(2) | 238(1) | 763(2) | 344(6) | 251(2) | 186(1) | 781(2) |
| 10+ | 4226(92) | 5454(98) | 32940(99) | 42620(98) | 4978(91) | 12170(96) | 25384(97) | 42532(96) |
| **Number of social contacts** |  |  |  |  |  |  |  |  |
| High contact (4-5) | 1974(43) | 2587(46) | 17304(52) | 21865(50) | 2074(38) | 6056(48) | 13951(53) | 22081(50) |
| Moderate contact (2-3) | 2143(46) | 2547(46) | 13853(42) | 18543(43) | 2633(48) | 5592(44) | 10498(40) | 18723(42) |
| Low contact (0-1) | 501(11) | 453(8) | 2021(6) | 2975(7) | 756(14) | 1069(8) | 1743(7) | 3568(8) |
| **Number of social activities** |  |  |  |  |  |  |  |  |
| High participation (4-5) | 822(18) | 1033(18) | 7264(22) | 9119(21) | 769(14) | 2526(20) | 5912(23) | 9207(21) |
| Moderate participation (2-3) | 2494(54) | 3410(61) | 20613(62) | 26517(61) | 2941(54) | 7613(60) | 16224(62) | 26778(60) |
| Low participation (0-1) | 1296(28) | 1133(20) | 5275(16) | 7704(18) | 1650(30) | 2396(19) | 3722(14) | 7768(18) |
| **Anxiety** |  |  |  |  |  |  |  |  |
| No | 3825(83) | 4813(86) | 30708(93) | 39346(91) | 4299(79) | 11103(87) | 23941(91) | 39343(89) |
| Yes | 777(17) | 757(14) | 2434(7) | 3968(9) | 1018(19) | 1316(10) | 1628(6) | 3962(9) |
| **Family doctor in last 12 months** |  |  |  |  |  |  |  |  |
| No | 328(7) | 461(8) | 3065(9) | 3854(9) | 454(8) | 1057(8) | 2421(9) | 3932(9) |
| Yes | 4275(93) | 5119(92) | 30042(91) | 39436(91) | 4991(91) | 11630(91) | 23720(91) | 40341(91) |
| **Unmet need** |  |  |  |  |  |  |  |  |
| Yes | 640(14) | 648(12) | 2353(7) | 3641(8) | 921(17) | 1250(10) | 1558(6) | 3729(8) |
| No | 3962(86) | 4931(88) | 30770(93) | 39663(91) | 4518(83) | 11445(90) | 24595(94) | 40558(91) |
| **ED visit** |  |  |  |  |  |  |  |  |
| Yes | 1329(29) | 1409(25) | 6936(21) | 9674(22) | 1571(29) | 3081(24) | 5312(20) | 9964(22) |
| No | 3268(71) | 4168(75) | 26167(79) | 33603(77) | 3863(71) | 9604(76) | 20827(80) | 34294(77) |
| **Care received** |  |  |  |  |  |  |  |  |
| No care received | 363(8) | 324(6) | 1139(3) | 1826(4) | 443(8) | 640(5) | 847(3) | 1930(4) |
| Non-professional received | 287(6) | 252(5) | 948(3) | 1487(3) | 331(6) | 540(4) | 666(3) | 1537(3) |
| Professional received | 624(14) | 687(12) | 3392(10) | 4703(11) | 775(14) | 1593(13) | 2466(9) | 4834(11) |
| Both non-professional and professional received | 3344(72) | 4324(77) | 27697(83) | 35365(82) | 3913(72) | 9942(78) | 22214(85) | 36069(81) |
