# Supplementary material for: Single-item versus scale: Comparing respondent demographic, social, and health characteristics by measure of loneliness using the Canadian Longitudinal Study on Aging (CLSA) data
Source: PLoS One. 2026 Feb 4;21(2):e0341572. doi: 10.1371/journal.pone.0341572 (PMC12871960; doi:10.1371/journal.pone.0341572)
Supplement: S4 Table — (DOCX) [file pone.0341572.s004.docx]

S4 Table. Descriptive characteristics of discordant respondents to 3-item scale and single-item loneliness measure (moderately lonely).

| **Variables** | **Responded that they were severely lonely (scale) and moderately lonely (single item)** | **Responded that they were not lonely (scale) and moderately lonely (single item)** |
| --- | --- | --- |
|  |  |  |
|  | **N (%)** | **N (%)** |
| **Total** | 1722 | 1259 |
| **Age** |  |  |
| 46-55 | 340(20) | 239(19) |
| 56-65 | 623(36) | 422(34) |
| 66-75 | 425(25) | 322(26) |
| 76-85 | 274(16) | 235(19) |
| 86+ | 60(4) | 41(3) |
| **Age (Mean, SD)** | 65.3(10) | 65.9(11) |
| **Age median (Q1-Q3)** | 64(57-72) | 65(57-74) |
| **Gender** |  |  |
| Gender diverse | 2(0.1) | 2(0.2) |
| Women | 1009(59) | 673(54) |
| Men | 711(41) | 584(46) |
| **Education** |  |  |
| Less than university | 921(58) | 654(55) |
| University or higher | 671(42) | 527(45) |
| **Ethnicity** |  |  |
| All else | 118(7) | 59(5) |
| White | 1604(93) | 1200(95) |
| **Geographic region** |  |  |
| Rural | 139(8) | 125(10) |
| Urban | 1583(92) | 1134(90) |
| **Income** |  |  |
| <$20,000 | 190(12) | 59(5) |
| $20,000-<$50,000 | 544(35) | 286(25) |
| $50,000+ | 837(53) | 795(70) |
| **Marital status** |  |  |
| Single, never married | 330(19) | 105(8) |
| Divorced/separated | 395(23) | 139(11) |
| Married/common law | 704(41) | 844(67) |
| Widowed | 292(17) | 170(14) |
| **Living alone** |  |  |
| No | 918(53) | 936(74) |
| Yes | 804(47) | 323(26) |
| **Number of chronic conditions** |  |  |
| <4 | 1200(70) | 997(79) |
| 4+ | 522(30) | 262(21) |
| **Functional impairment** |  |  |
| None | 1283(77) | 1040(86) |
| Mild/moderate/severe/total | 374(23) | 173(14) |
| **Self-rated mental health** |  |  |
| Poor | 43(3) | 13(1) |
| Fair/Good/Very Good/Excellent | 1678(97) | 1245(99) |
| **Number of depressive symptoms** |  |  |
| <10 | 55(3) | 23(2) |
| 10+ | 1667(97) | 1236(98) |
| **Number of social contacts** |  |  |
| High contact (4-5) | 686(40) | 661(53) |
| Moderate contact (2-3) | 850(49) | 521(41) |
| Low contact (0-1) | 186(11) | 77(6) |
| **Number of social activities** |  |  |
| High participation (4-5) | 261(15) | 270(22) |
| Moderate participation (2-3) | 1001(58) | 799(64) |
| Low participation (0-1) | 457(27) | 185(15) |
| **Anxiety** |  |  |
| No | 1422(83) | 1132(90) |
| Yes | 296(17) | 125(10) |
| **Family doctor in last 12 months** |  |  |
| No | 138(8) | 100(8) |
| Yes | 1581(92) | 1157(92) |
| **Unmet need** |  |  |
| Yes | 274(16) | 97(8) |
| No | 1445(84) | 1160(92) |
| **ED visit** |  |  |
| Yes | 474(28) | 297(24) |
| No | 1243(72) | 961(76) |
| **Care received** |  |  |
| No care received | 123(7) | 57(5) |
| Non-professional received | 95(6) | 40(3) |
| Professional received | 216(13) | 127(10) |
| Both non-professional and professional received | 1285(75) | 1033(82) |
